# The application of a lateral flow immunographic assay to rapidly test for dexamethasone in commercial facial masks

**DOI:** 10.1007/s00216-019-01948-2

**Published:** 2019-07-24

**Authors:** Min Wang, Liqun Guo, Miao Yu, Hua Zhao

**Affiliations:** College of Science, Beijing Technology and Business University, Beijing, 102488 China

**Keywords:** Dexamethasone, Lateral flow immunographic assay, Cosmetics, Field test

## Abstract

Dexamethasone (DE) is a synthetic glucocorticoid that is frequently added to cosmetic products for its good short-term effects, especially in facial masks, but long-term use is hazardous to the health. The abuse of DE in whitening and acne cosmetic products is currently a serious problem in China. It is necessary to establish a rapid method of detecting illegal DE addition in cosmetics. In the present study, a monoclonal antibody (mAb) against DE, 2D5-3D12, was developed that displayed cross-reactivities of 124.5%, 38.8%, 6.7%, 0.9%, 1.1%, 1.82%, and 2.39% with prednisolone, betamethasone, prednisone, beclomethasone, hydrocortisone, triamcinolone, and flumetasone, respectively. A colloidal gold-based lateral flow immunographic assay based on mAb 2D5-3D12 was established and used to determine the DE contents of commercial facial masks. The indicator range of the immunographic assay for DE was 100–200 ng/mL, and the results were consistent with those afforded by LC-MS. This novel method provides the advantages of simple sample treatment, a user-friendly procedure, and rapid detection.

**Electronic supplementary material:**

The online version of this article (10.1007/s00216-019-01948-2) contains supplementary material, which is available to authorized users.

**Figure:**
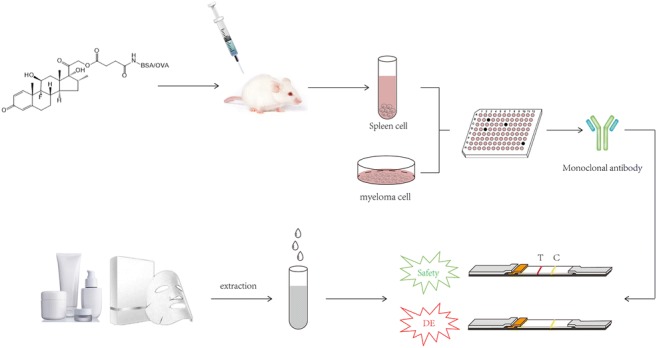
Graphical abstract

## Introduction

Immunosuppressive agents, including glucocorticoids, are widely used to control skin inflammation. However, these drugs may induce severe and partially irreversible adverse effects, such as skin atrophy, striae distensae, telangiectasia, and—rarely—hypothalamic-pituitary-adrenal axis suppression [1]. Dexamethasone (DE) is a synthetic glucocorticoid that improve skin conditions such as eczema and may even alleviate severe psoriasis caused by skin inflammation [2, 3]. However, the long-term use of DE in large doses can lead to dependence, dilation of blood vessels, red spots in the skin, and numerous other side effects. In order to enhance the effects of their products, some cosmetics companies illegally add glucocorticoids to products such as facial masks and creams, which results in rapid whitening and skin rejuvenation. In recent years, due to the widespread use of topical hormonal preparations and unregulated cosmetics containing hormones, the incidence of facial glucocorticoid-dependent dermatitis, which seriously affects physical and mental health, has increased such that it is now the fifth most frequent outpatient skin condition after eczema, psoriasis, acne, and urticaria [4].

There are regulations prohibiting the addition of 41 glucocorticoids such as DE to cosmetics. According to the Safety and Technical Standards for Cosmetics (no. 268, 2015), issued by the National Medical Products Administration, glucocorticoids are prohibited from being used as components or raw materials in cosmetics. However, in recent years, a large number of illegal cosmetics have been detected in cosmetics.

To standardize the market and protect consumers’ legitimate rights and interests, it is necessary to establish an efficient and sensitive method for detecting DE in cosmetics. The most commonly used method of detecting DE in cosmetics is mass spectrometric analysis [5, 6], but this method is inconvenient and cannot be used for real-time high-throughput detection in the field. In China, the LOD achieved with the national standard method of detecting glucocorticoids (National Standard of the People’s Republic of China GB/T 24800.2–2009)—a method based on LC-MS-MS and TLC—was 0.03 μg/g, and the LOQ was 0.1 μg/g.

The colloidal gold strip test method is convenient, simple, and easy to perform, especially when used for rapid detection on-site, and the results obtained are easy to interpret. Colloidal gold immunoassay strips are used in various fields (e.g., for disease and drug detection [7–11]), but applications of these strips in skin care products are scarce; only strips for detecting ricin [12] and mulberroside A [13] have been reported. In this study, we developed a specific and sensitive monoclonal antibody (mAb) against DE. A lateral flow immunographic assay (LFIA) that utilizes this mAb was then developed to enable rapid screening for DE in cosmetics. Finally, the LFIA was used to detect the DE levels in commercial facial masks containing either high, moderate, or low levels of DE, and the results were consistent with those obtained by liquid chromatography–mass spectrometry (LC-MS).

## Materials and methods

### Reagents and buffers

The reagents bovine serum albumin (BSA), ovalbumin (OVA), Freund’s complete adjuvant and incomplete adjuvant, goat anti-mouse IgG, *N*-hydroxysuccinimide (NHS), and *N*,*N*′-dicyclohexylcarbodiimide (DCC) as well as the immunoglobulin isotype test kit were purchased from Sigma–Aldrich Chemical Co. (Shanghai, China). Colloidal gold particles (30 nm), conjugate pads, polyvinyl chloride (PVC) plates, nitrocellulose (NC) membrane absorption pads, and sample pads were purchased from Jieyi Biotech Co. (Shanghai, China). Dexamethasone, prednisolone, betamethasone, prednisone, beclomethasone, hydrocortisone, triamcinolone, flumetasone, cortisone, and triamcinolone acetonide were purchased from Heowns Biochem Tech Co. (Tianjin, China). All other reagents were purchased from Beijing Chemical Reagents Co. (Beijing, China).

### Hapten synthesis and conjugate preparation

**Fig. 1:**
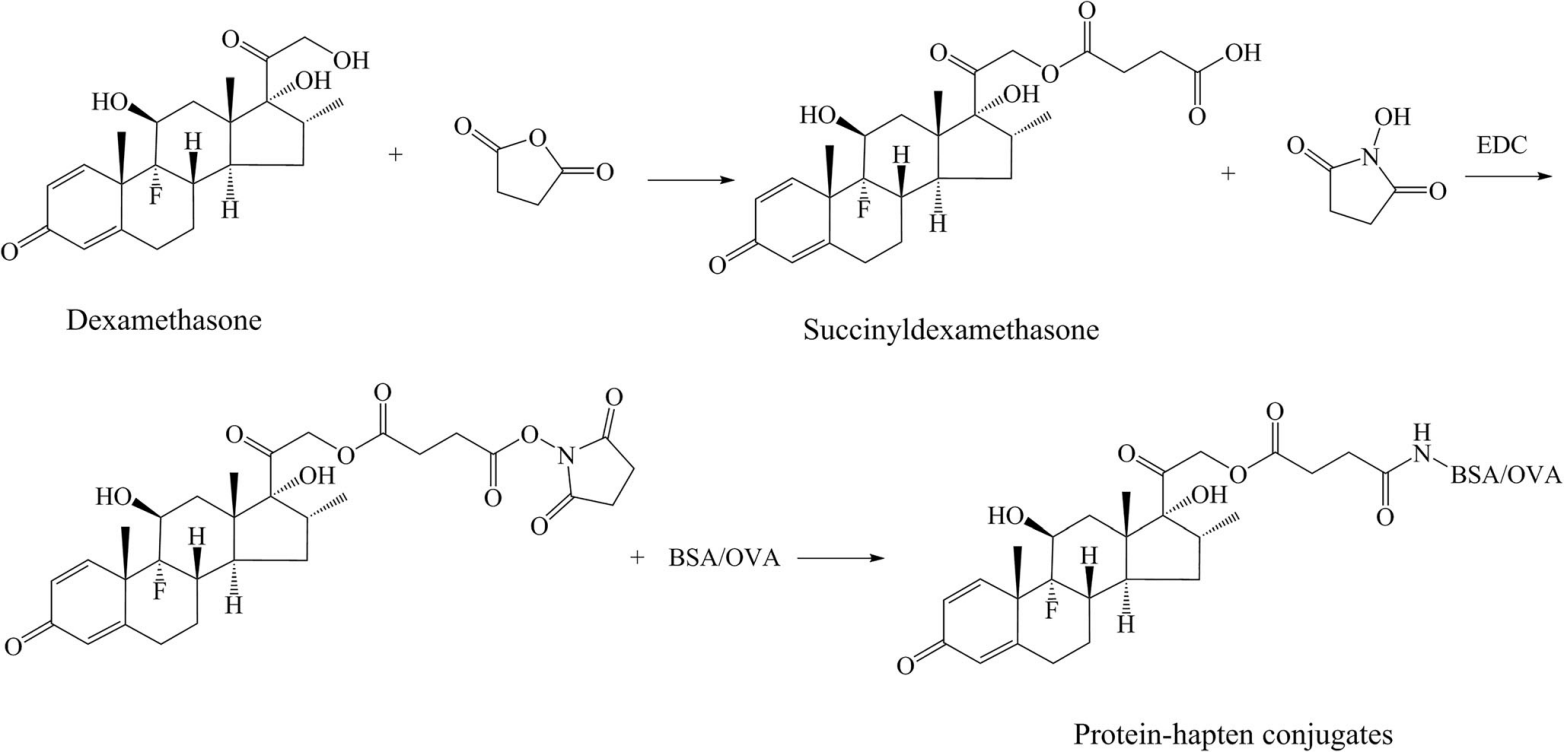
Preparation of the DE hapten and the protein–hapten conjugate

To prepare the hapten, 100 mg of the DE standard were dissolved in 5 mL of anhydrous pyridine and then 31 mg of succinic anhydride were added to the solution. The mixture was stirred overnight at RT and then subjected to rotary evaporation under reduced pressure at 35 °C to dryness. The residue was purified with a silica gel column (30 g, 25 cm) using a petroleum ether-ethyl acetate (1/1, v/v) mixture as the elution system to afford the hapten as a yellow liquid (22 mg, 18%). HRMS: *m*/*z* calcd for C_26_H_33_FO_8_: [M + H^+^] 493.2232, found 493.2230 (Fig. 1).

The hapten was conjugated with BSA and OVA by the carbodiimide method to produce the an immunogen (DE-BSA) and coating antigen (DE-OVA) (Fig. 1). Briefly, 16 mg of DCC and 9 mg of NHS were added to 9.8 mg of the hapten in 0.4 mL of DMF. The solution was stirred overnight at RT. After centrifugation, the supernatant of the reaction mixture was added dropwise to 26 mg of BSA or 39 mg of OVA dissolved in 5 mL of 0.01 M phosphate-buffered saline (PBS containing 0.9% NaCl, pH 7.5) and stirred overnight at 4 °C. The conjugates were dialyzed against PBS at 4 °C. After dialysis, the DE-BSA and DE-OVA conjugates were stored at −20 °C.

### Development of a monoclonal antibody against DE

The protocols used for immunization, fusion, antibody production, and purification were the same as those described previously [14]. Briefly, six 7-week-old female BAL b/c mice were immunized with DE-BSA (0.1 mg) in Freund’s complete adjuvant and were boosted with the DE-BSA immunogen (0.1 mg) in Freund’s incomplete adjuvant at 2-week intervals. After the fourth immunization, the spleen cells of the best mouse were fused with SP2/0 cells. Seven days after fusion, the supernatant was tested and cloned by limiting dilution. The clone with the highest antibody titer and good sensitivity in the culture supernatant was expanded in mice, leading to the production of ascites fluid. The required monoclonal antibody was then purified from the ascites by ammonium sulfate precipitation.

The experiments involving animals carried out in this work were performed in strict accordance with the standards described in the Guide for the Care and Use of Laboratory Animals (National Research Council Commission on Life Sciences, 1996 edition). All animal treatment procedures were performed in China Agricultural University and approved by the Animal Care Committee of China Agricultural University.

### Antibody specificity

The immunoglobulin isotype of the mAb was evaluated using the test kit according to the instructions of the manufacturer (Sigma–Aldrich). The specificity of the mAb against DE was evaluated by cross-reactivity (CR). The IC50 of each structural analog of DE was measured with a previously reported icELISA [15], and the CR was calculated using the following formula:

$$ \mathrm{CR}\left(\%\right)=\frac{\mathrm{IC}50\ \mathrm{of}\ \mathrm{DE}}{\mathrm{IC}50\ \mathrm{of}\ \mathrm{other}\ \mathrm{compound}}\times 100 $$

### Development of a colloidal gold-based LFIA

Using the antibody against DE, a LFIA for DE was developed using a method described previously [16]. Briefly, 5 mL of colloidal gold (pH 7.8) were added to 40 μL of mAb (1.0 mg/mL aqueous solution) and stirred for 20 min. Then 200 μL of 10% (w/v) BSA were added to stabilize the gold-Ab particles and this mixture was stirred for another 15 min before being centrifuged at 10,000 rpm for 10 min. After discarding the supernatant, the gold-Ab conjugate was redissolved in 1 mL of Na_2_HPO_4_-KH_2_PO_4_ buffer (pH 7.4, 0.01 M) and immobilized on the conjugate pad. NC membrane was pasted onto the center of the PVC plate, and then 0.5 mg/mL of goat anti-mouse IgG and 1 mg/mL of DE-OVA were immobilized on the NC membrane at 1 μL/cm as the control line (C line) and the test line (T line), respectively. The two lines were separated by a distance of 0.5 cm. To assemble the LFIA, the conjugate pad and sample pad were sequentially pasted downstream of the NC membrane, while the absorption pad was pasted upstream. Each pad was pasted such that there was a 0.2-cm overlap with the adjacent pad. The individual LFIAs were cut to a width of 3 mm, then placed in plastic container, sealed in aluminum foil, and stored at 4 °C.

### Evaluating the range of the indicator

DE was dissolved in methanol to a concentration of 1 mg/mL as stock solution. A series of standard solutions (10, 25, 50, 100, 200, 500, and 1000 ng/mL) were then prepared with the stock solution. Seventy microliters of the standard solution were pipetted onto the assay strips. After allowing the colors of the T and C lines to develop for 10 min, the lowest concentration range at which the T line was not visible was defined as the indicator range. Each sample was analyzed in triplicate.

### Reproducibility and stability of the LFIA

The reproducibility of the LFIA was studied by applying it to facial mask samples. Assays were repeated three times each day and conducted for three successive days. To determine the performance of the assay after storage, the LFIA was stored at 4, 25, or 37 °C for 1 week, 1 month, or 6 months. The range of the indicator for DE was re-evaluated after storage.

### Detection of DE in commercial facial masks by LFIA

Commercial facial masks were purchased from online and physical stores in Beijing (see Table S1 in the “Electronic supplementary material,” ESM). One gram of each sample was weighed and transferred to a 10-mL tube. The DE was extracted using 5 mL of acetonitrile and saturated salt water (2/3, v/v) as the extraction reagent and applying ultrasonic waves for 10 min. The extracts were centrifuged at 5000 r/min for 10 min and the supernatant was transferred to a new tube. The supernatant was evaporated to dryness under nitrogen, and the extract was re-dissolved in PBS for analysis by LFIA.

### Detection of DE in commercial facial masks by LC-MS

For LC-MS detection, the extraction was performed as above. Thirty-six milliliters of ddH_2_O, 0.2 mL of potassium ferrocyanide, and 0.2 mL of zinc acetate were added to the supernatant of the extract. After thorough vortexing, the mixture was centrifuged at 5000 r/min for 10 min. The supernatant was purified via solid-phase extraction (using a n Oasis HLB cartridge). The eluent was evaporated to dryness under nitrogen and 1 mL of 50% methanol was added to dissolve the target, which was then subjected to LC-MS according to the Chinese national standard method GB/T 24800.2–2009. Each sample was analyzed in triplicate.

## Results and discussion

### Hapten synthesis and conjugate preparation

A suitable mAb to use in a LFIA test for DE was developed. The DE hapten and carrier protein were conjugated by the carbodiimide method to serve as the immunogen and coating antigen, respectively. The conjugation of DE-BSA and DE-OVA was confirmed by UV-vis spectroscopy. The results showed that the hapten was successfully coupled with the carrier protein. The molar ratios of DE to BSA and DE to OVA were estimated to be 5:1 and 4:1, respectively.

### Development of the monoclonal antibody against DE

Three days after the fourth boost immunization, spleen cells were collected from the mice with the best antiserum titer (1:8000, the antiserum dilution that gave an absorbance of 1.0 under noncompetitive assay conditions) and inhibition of 1000 ng/mL of DE (86%) for in vitro hybridoma cell production. After cloning the hybridoma cell lines using limiting dilution, the clone with the best inhibition of DE (100 ng/mL, 75%),denoted 2D5-3D12, was expanded for ascites production. The titer for the ascites was 1.6 × 10^4^. The mAb was confirmed to be an IgG2a isotype.

### Antibody specificity

The CR of the mAb 2D5-3D12 was tested via specificity analysis. The results are summarized in Table 1, which shows the structures, IC50 values, and CR values from icELISA for prednisolone, betamethasone, prednisone, beclomethasone, hydrocortisone, triamcinolone, flumetasone, cortisone, and triamcinolone acetonide. According to the test results for recent years reported by the National Quality Supervision and Administration Bureau, dexamethasone and clobetasol propionate are the glucocorticoids that are most commonly added to cosmetics, due to their low cost and ready availability. Prednisolone had never been detected in Chinese cosmetics. Thus, although the cross-reactivity of prednisolone was 124.5%, it would not interfere in the detection of DE in cosmetics because it is never present. The cross-reactivities of prednisone, beclomethasone, hydrocortisone, triamcinolone, and flumetasone were approximately 6.7%, 0.9%, 1.1%, 1.82%, and 2.39%, respectively. No competitive inhibition was observed when up to 10,000 ng/mL cortisone and triamcinolone acetonide were present.

**Table 1:**
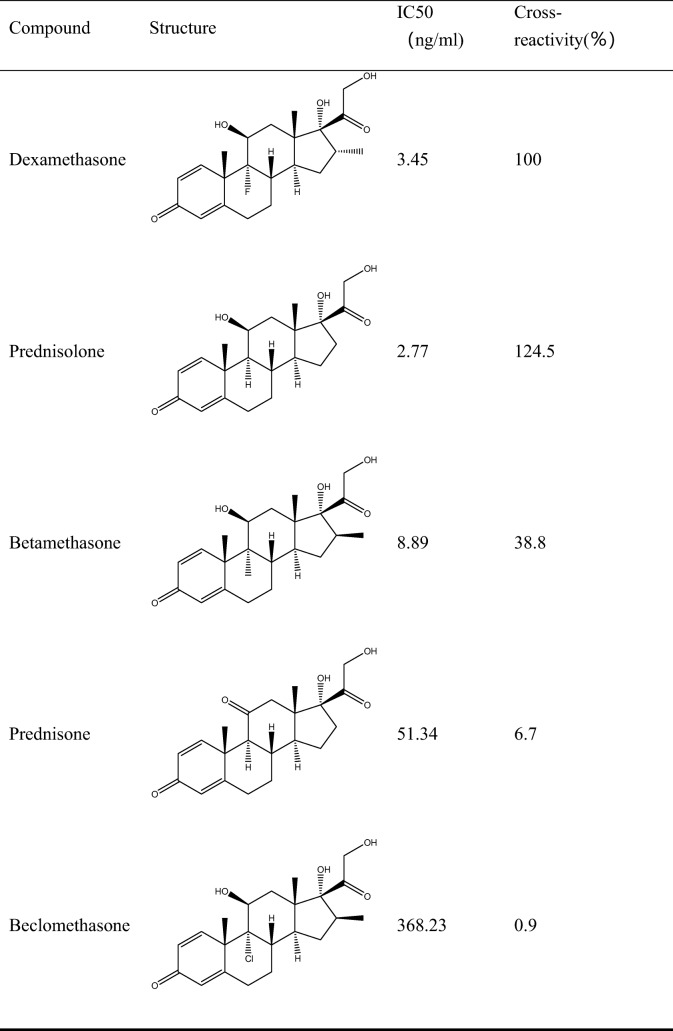

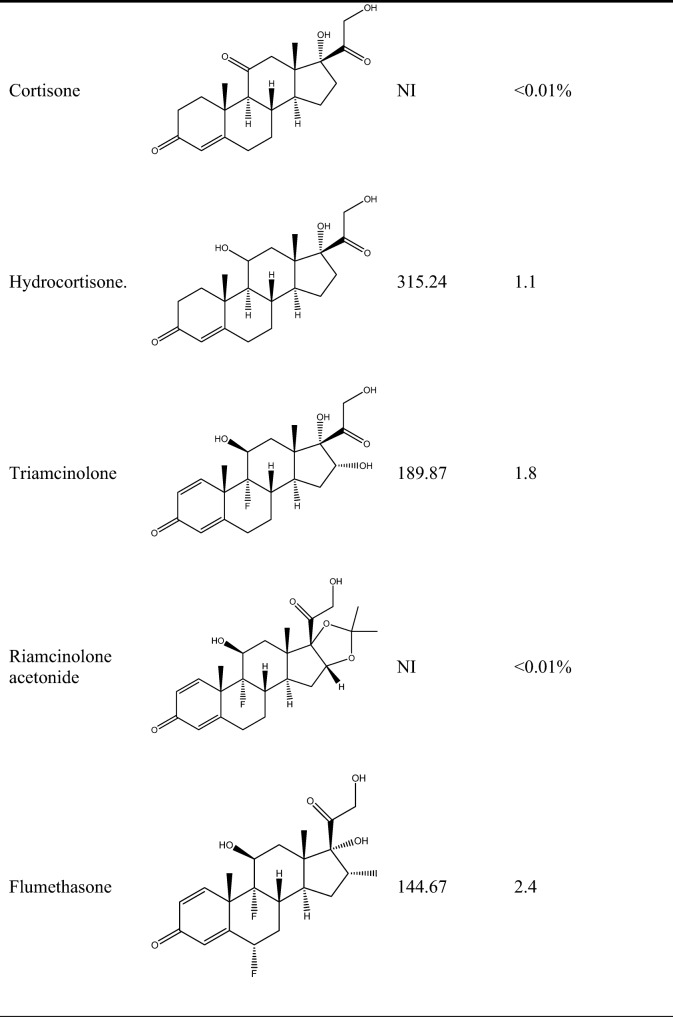
Cross-reactivities of DE and its analogs

### Determination of the indicator range of the LFIA

**Fig. 2:**
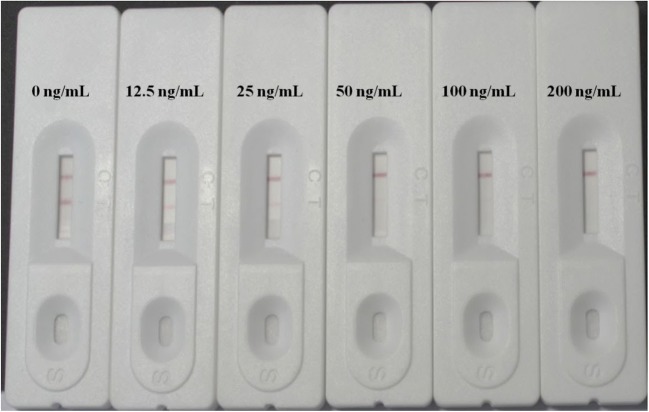
Photographs of LFIAs showing how the color intensity of the T line changes with the concentration of DE in PBS buffer (Na_2_HPO_4_–KH_2_PO_4_, 0.01M, pH 7.5). The indicator range for DE was 10–200 ng/mL

The mAb 2D5-3D12 was applied to develop a colloidal gold-based LFIA. The assay utilized competitive and qualitative determination. As shown in Fig. S1 of the ESM, the sample to be tested for DE was added to the sample pad of the test strip. The liquid reagent then migrated by capillary action through the conjugate pad. The colloidal gold–Ab conjugate re-dissolved and bonded with any DE present. After the liquid reagent had passed through the reaction zone, the color intensity of the T line was used to discern the level of DE present. A low level of DE was indicated by the presence of a bright T line, since a large amount of colloidal gold–Ab conjugate was bonded with DE-OVA at the T line. When there was a high level of the target, the conjugated particles were bound to the DE analyte and were therefore not captured at the T line, so the T line was colorless. A moderate level of DE was indicated by a faint T line. The control line containing the rabbit anti-mouse IgG captured the colloidal gold–Ab conjugate and turned bright red, whether the level of DE was high, moderate, or low. The optimum concentrations of DE-OVA, goat anti-mouse antibody, and colloidal gold–mAb 2D5-3D12 were found to be 1 mg/mL, 1 mg/mL, and 30 μg/mL, respectively. The concentration of colloidal gold–mAb present was calculated based on the concentration of antibody added when preparing the colloidal gold–mAb conjugate. The concentration dependence of the color intensity of the LFIA for standard DE in PBS (0.01 M, pH 7.5) is illustrated in Fig. 2. The indicator range of the LFIA for DE was found to be 10–200 ng/mL.

### Reproducibility and stability of the LFIA

To evaluate the reproducibility of the LFIA, two facial mask samples (A5 and B3, Table 3) that had already been analyzed by LC-MS were assayed using the LFIA three times each day for three days. The resulting color development in the LFIA is shown in Fig. S2 of the ESM. There were consistent visual chromatographic bands across the nine assays.

The storage stability of the LFIA was evaluated at three different temperatures. There was no change in the indicator range of the LFIA after 6 months of storage at 4 °C or after 1 month of storage at 25 °C (Table 2). However, the sensitivity of the LFIA was severely affected, making the assay unusable, after one week at 37 °C.

**Table 2:** The storage stability of the LFIA

Storage conditions	Indicator range (ng/mL)			
	Day 0	After 1 week	After 1 month	After 6 months
4 °C	10–200	10–200	10–200	10–200
25 °C	10–200	10–200	10–200	–
37 °C	10–200	–	–	–

### Analysis of the DE contents of commercial facial masks

**Fig. 3:**
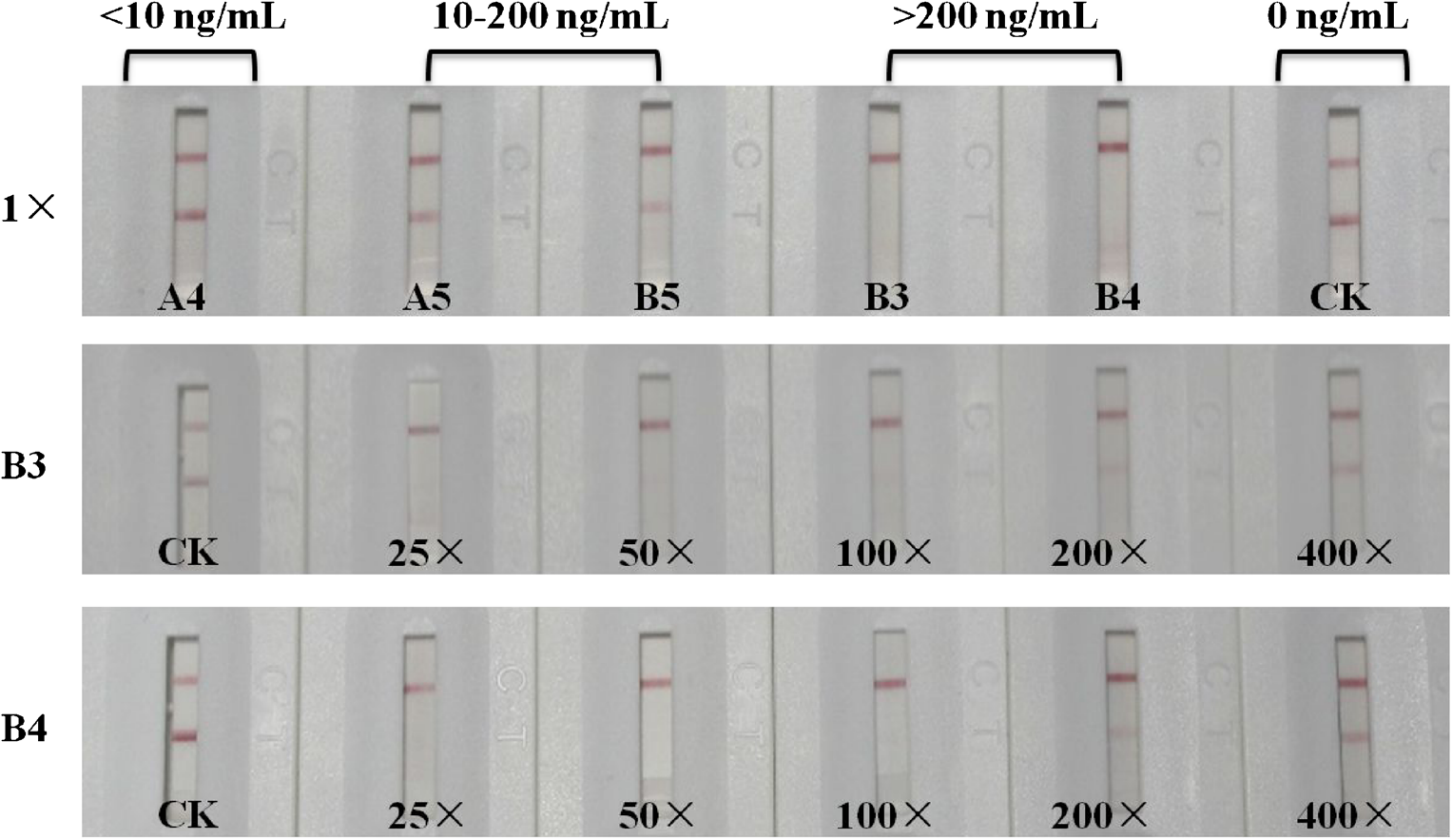
LFIA analysis of DE in commercial facial mask samples (A4, A5, B3, B4, and B5, which was undiluted, 1×). Samples B3 and B4, which contained high levels of DE, were diluted 25-, 50-, 100-, 200-, and 400-fold for re-detection. The results showed that A4 contained low levels of DE (< 10 ng/mL), while A5 and B5 contained moderate levels of DE (10–100 ng/mL). B3 and B4 had high levels of DE (4000–8000 ng/mL). DE was not detected in any of the other samples. PBS buffer (Na_2_HPO_4_–KH_2_PO_4_, 0.01 M, pH 7.5) containing no DE was applied as a control (*CK*)

The DE contents of commercial facial masks were determined using the LFIA. Forty-two samples were found to contain low levels of DE (< 10 ng/mL), while two samples, A4 and B5, contained moderate levels of DE (10–100 ng/mL). Two other samples, B3 and B4, contained high levels of DE (4000–8000 ng/mL) (Fig. 3). LC-MS was applied to confirm the DE contents of these products. The DE concentrations in the solutions determined by LC-MS agreed well with those determined by the LFIA (Table 3), demonstrating the reliability of the LFIA. The results showed that the assay had a similar detection range to LC-MS, but the pretreatment required for the LFIA was much simpler, the assay permitted the rapid analysis of large numbers of samples, and the data from the LFIA was provided in a user-friendly manner. To simplify the pretreatment process, we diluted the nutritive medium of the mask with PBS directly, and this diluted solution was then analyzed using the LFIA. When the mask medium was diluted more than fivefold, most of the interference from the mask was eliminated, making this approach especially suitable for field tests. However, the interference from the mask depends on the manufacturer of the mask and the particular product line considered, so it is important to analyze a few samples using instrument-based methods.

**Table 3:** Comparison of LC-MS and the LFIA assay for the determination of DE in commercial facial masks

Sample(s)	Determined DE content (ng/mL)	
	LC-MS	LFIA
B3	5778.1 ± 2.7	4000–8000
B4	5963.6 ± 6.5	4000–8000
B5	22.4 ± 0.3	10–100
A4	ND	<10
A5	ND	10–100
Others	ND	<10

## Conclusions

Cosmetics is the category of the global fast-moving consumer goods (GFMCG) market that exhibits the most stable growth. According to the National Food and Drug Administration website, from 2016 to 2018, glucocorticoids such as DE were illegally added to many batches of cosmetic products. In China, in addition to products sold in regular supermarkets, the areas hardest hit by the illegal addition of glucocorticoids to cosmetics include e-commerce, micro businesses, and beauty salon products.

Consumers can achieve rapid whitening skin rejuvenation by using whitening and acne cosmetic products containing DE and other glucocorticoids. However, the long-term use of cosmetics containing glucocorticoids may cause dark spots, shrinkage and thinning of the facial skin, and hormone-dependent dermatitis. Cosmetic regulations of the European Union (regulation (EC) 1223/2009) and China clearly stipulate that the presence of glucocorticoids, estrogens, androgens, progesterone, and other hormones in cosmetic components is prohibited substances.

At present, most of the methods used to detect prohibited substances in cosmetics are based on high-performance liquid chromatography, HPLC-MS, etc., and therefore require large instruments that must be operated by professionals. Also, the utility of current detection methods is limited by the experimental conditions and sample preparation required.

We therefore used a newly selected mAb, 2D5-3D12, to develop and optimize a LFIA for the semiquantitative analysis of DE in cosmetics. The indicator range of the LFIA for DE was 10–200 ng/mL. The semiquantitative analysis of DE in commercial facial masks with the LFIA yielded results consistent with those afforded by LC-MS, which indicated that the developed LFIA was a reliable method for the semiquantitation of DE. Storage tests showed that the indicator range for DE remained unchanged after 6 months of storage at 4 °C or 1 month at ambient temperature. Overall, the results indicated that this new immunoassay could be employed for the fast and convenient determination of DE in commercial facial masks.

LFIAs have been widely used in food, agricultural product, and environmental testing, and are simple, inexpensive, and fast. The aim of the present work was to develop a LFIA method for detecting DE in cosmetic samples that could be used for on-site sampling by professionals as well as on a daily basis by consumers. The developmeny of this method may also assist with the monitoring and regulation of illegal additives in Chinese cosmetics.

## Electronic supplementary material

ESM 1 (PDF 1.29 mb)
