# Supplementary material for: The application of a lateral flow immunographic assay to rapidly test for dexamethasone in commercial facial masks
Source: Anal Bioanal Chem. 2019 Jul 24;411(22):5703–10. doi: 10.1007/s00216-019-01948-2 (PMC6704111; doi:10.1007/s00216-019-01948-2)
Supplement: Supplementary file 1 — (PDF 1.29 mb) [file 216_2019_1948_MOESM1_ESM.pdf]

Analytical and Bioanalytical Chemistry

Electronic Supplementary Material

The application of a lateral flow immunographic assay to rapidly test for dexamethasone in commercial facial masks

Min Wang, Liqun Guo, Miao Yu, Hua Zhao

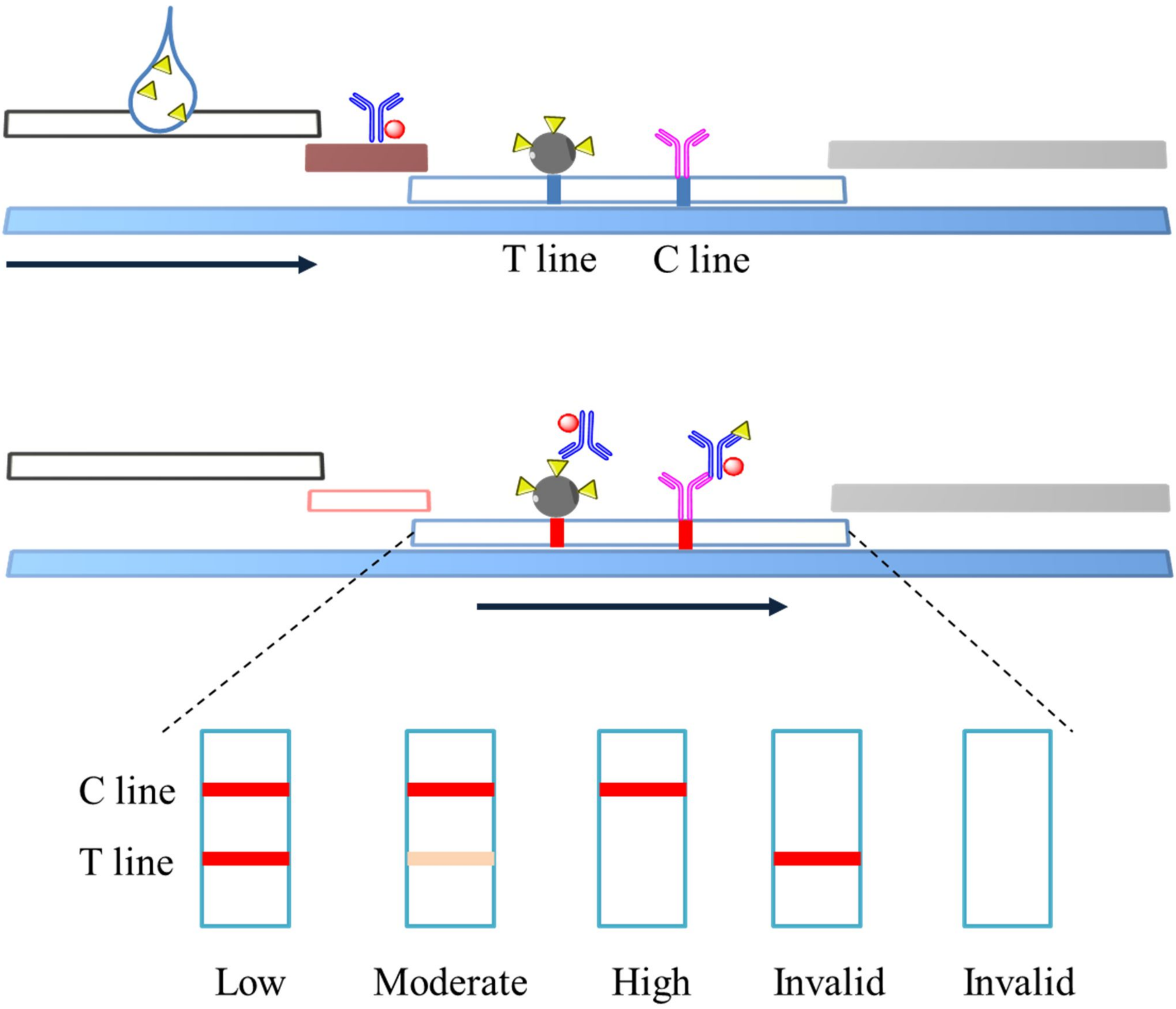

Fig. S1 Schematic diagram of LFIA for DE. The C line and T line were coated with goat anti-mouse IgG and DE-OVA, respectively. Gold-labeled mAb was dispensed on the conjugate pad. The analytical solution was spiked to the sample pad and pass through the conjugate pad, T line and C line by capillary action

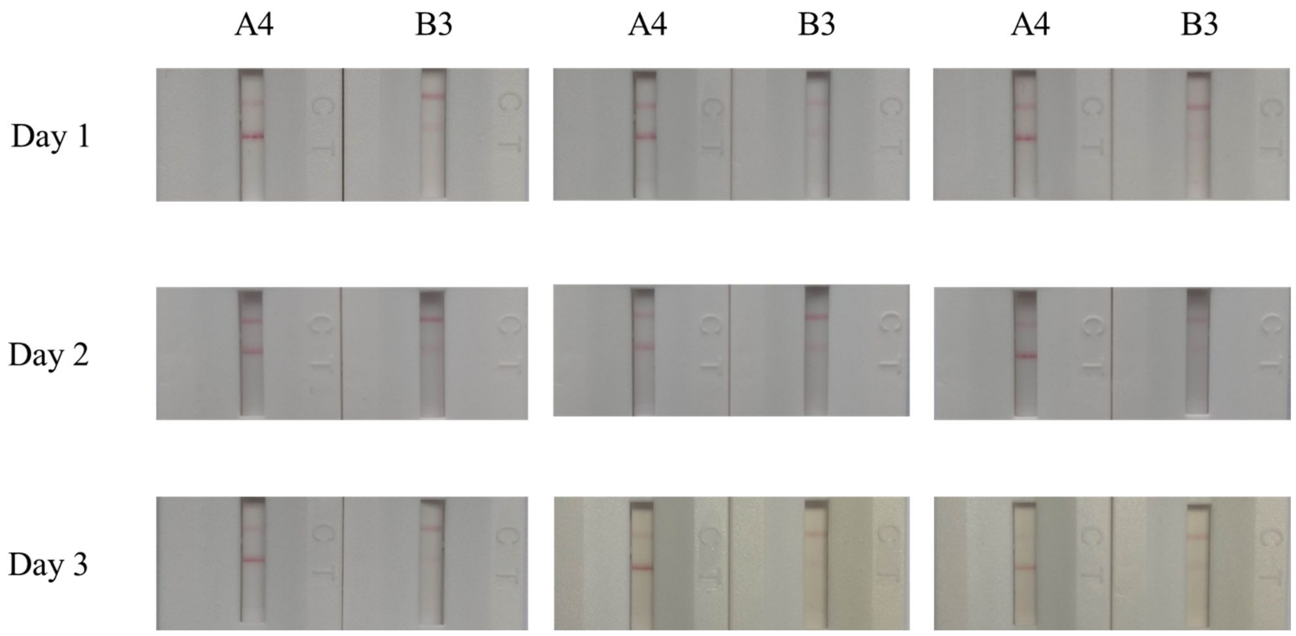

Fig. S2 The reproducibility evaluation of the LFIA. The sample A4 (Left) and B3 (200 fold dilution, Right) was used to determine the DE content for 3 times per day, and repeated 3 days. Each assay was performed in triplicate, and one of which was digital imaging

Table S1 The information of the commercial facial masks

No.	Brand	Origin	marketing channel
1	Gialen	Guangzhou, Guangdong	Physical stores
2	2N	Guangzhou, Guangdong	Physical stores
3	Pechoin	Shanghai	Physical stores
4	Hada Labo	Zhongshan, Guangdong	Physical stores
5	OLAY	Shanghai	Physical stores
6	Hada Labo	Zhongshan, Guangdong	Physical stores
7	TONYMOLY	Hangzhou, Zhejiang	Physical stores
8	Herborist	Shanghai	Physical stores
9	O`LIYE	Guangzhou, Guangdong	Physical stores
10	INOHERB	Shanghai	Physical stores
11	OLAY	unknown	Physical stores
12	RONYME	Guangzhou, Guangdong	Physical stores
13	Tongrentang	Beijing	Physical stores
14	Jinronghua	Shanghai	Physical stores
15	2N	Guangzhou, Guangdong	Physical stores
16	2N	Guangzhou, Guangdong	Physical stores
17	MG	Guangzhou, Guangdong	Physical stores
18	Pechoin	Shanghai	Physical stores
19	INOHERB	Shanghai	Physical stores
A1	8 cup water	Guangzhou, Guangdong	Online store
A2	Huanyancao	Guangzhou, Guangdong	Online store
A3	Hanhuo	Guangzhou, Guangdong	Online store

A4	8 cup water	Guangzhou, Guangdong	Online store
A5	Huanyancao	Guangzhou, Guangdong	Online store
A6	Huanyancao	Guangzhou, Guangdong	Online store
A7	Huanyancao	Guangzhou, Guangdong	Online store
A8	Babiqi	Guangzhou, Guangdong	Online store
A9	Babiqi	Guangzhou, Guangdong	Online store
A10	Babiqi	Guangzhou, Guangdong	Online store
A11	Hanhuo	Guangzhou, Guangdong	Online store
A12	Babiqi	Guangzhou, Guangdong	Online store
A13	BIBOO	Guangzhou, Guangdong	Online store
A14	JUNYISHENG	Guangzhou, Guangdong	Online store
A15	BIBOO	Guangzhou, Guangdong	Online store
A16	BIBOO	Guangzhou, Guangdong	Online store
A17	JUNYISHENG	Guangzhou, Guangdong	Online store
B1	Biquanhua	Guangzhou, Guangdong	Online store
B2	Oadmire	unknown	Online store
B3	Hanmei	Shanghai	Online store
B4	Hanmei	Shanghai	Online store
B5	MG	Guangzhou, Guangdong	Online store
B6	Qiaofuquan	Foshan, Guangdong	Online store
B7	MYTCF	Hangzhou, Zhejiang	Online store
B8	BANOVA	Hong Kong	Online store
B9	BOVEY	unknown	Online store
B10	Babycoco	Guangzhou, Guangdong	Online store
